# Editorial: Impact of immunotherapy in lung cancer

**DOI:** 10.3389/fonc.2022.1083524

**Published:** 2022-11-11

**Authors:** Marion Ferreira, Karen L. Reckamp

**Affiliations:** ^1^ Department of Pneumology and Respiratory Functional Exploration, University Hospital of Tours, Tours, France; ^2^ INSERM, Centre d’Etude des Pathologies Respiratoires, Tours, France; ^3^ Department of Medicine, Cedars Sinai Medical Center, Los Angeles, CA, United States

**Keywords:** immunotherapy, lung cancer, checkpoint inhibition, biomarkers, non-small cell lung cancer, small-cell lung cancer

Since the approval of the first immune checkpoint inhibitors (ICI), the treatment paradigm of lung cancer and survival outcomes have been profoundly altered (1, 2). In addition to the characteristics of the tumor cell, the growth and metastatic potential of cancer are also dependent on interactions with the immune system. Checkpoints allow the immune system to avoid unwanted damage to adjacent tissues possibly caused by activated T cells. In cancer, T-cells primed to respond to tumor cells are exposed continuously to tumor antigens within the malignancy, which may result in upregulation of multiple inhibitory receptors, culminating in decreased cytotoxic activity against tumor cells. This T-cell exhaustion can be overcome by modulating the inhibitory pathways with antagonist antibodies, ICI (3). We present in this issue, through a few articles selected and seven studies detailed, a summary of the immunotherapy history in lung cancer, its hopes for efficacy improvement and its challenges for the future.

The immunotherapy revolution started in pretreated advanced non-small cell lung cancer (NSCLC) with several authorizations for anti-programmed-death 1 (PD-1) (1, 2, 4) and anti-programmed-death ligand 1 (PD-L1) (5) ICI as a second line option. Quickly, ICI became a cornerstone of first line NSCLC management, either as a single modality (6, 7) or in association with chemotherapy (8, 9) but also with anti-cytotoxic T-lymphocyte-associated protein 4 (CTLA-4), leading to improvements in survival. This was followed by clinical benefit in the first line treatment of small cell lung cancer (SCLC), in association with chemotherapy (10, 11). Most of the immunotherapy clinical trials that have led to the marketing of various ICIs have been carried out in international populations. It is still necessary to keep in mind the variability in the patients’ characteristics, in particular when they come from different continents. Interestingly, Liu et al. evaluated the applicability of the ORIENT-11 immunotherapy study, performed only in China, in a Western population. All these advances in lung cancer treatment options resulted in, among other things, a decrease in population-level mortality from lung cancer (12).

In the management of advanced NSCLC and SCLC, immunotherapy has brought significant improvements in survival and quality of life for patients. Studies showed a significantly improved survival in patients after ICI implementation in the lung cancer treatment landscape (13–15). Thus, the five-year survival rate in all-stages NSCLC increased from 14% to 23.7% (American Lung Association, State of lung cancer, 2021). This increase in patient survival will further improve with indications expansion for immunotherapy in the curative setting. ICI are used as consolidation after concomitant chemoradiotherapy in NSCLC (16) but new indications is developing in the neoadjuvant and adjuvant settings (17, 18). For example, chemo-immunotherapy was approved in 2022 for early-stage NSCLC (19) and many other studies are in progress to specify the modalities of ICI use in the perioperative setting. Thus, Zhang et al. showed interesting data about perioperative outcomes of video-assisted thoracoscopic surgery versus open thoracotomy after neoadjuvant chemoimmunotherapy in resectable NSCLC.

Today, ICI are indicated for all stages of NSCLC and in advanced SCLC. Moreover, patients with rare histological types of lung cancer, for whom treatment options are even more limited, may benefit from ICI. Indeed, Xiao et al. showed that in pulmonary lymphoepithelioma-like carcinoma, first-line treatment with chemoimmunotherapy would be the best option in advanced stages. But additional issues and questions remain unresolved. Although revolutionary, too few patients seem to derive long-term benefit from immunotherapy. We need to better understand ICI mechanisms of primary and acquired resistance in order to personalize the management of all patients treated with ICI for lung cancer, NSCLC or SCLC, with or without oncogenic addiction, regardless of PD-L1 expression or disease stage. For example, a review by Titmarsh et al. analyzed current knowledge, prognostic significance and immunoregulatory role of c-MET-HGF axis in NSCLC.

In the future, further advances are needed. Robust and predictive biomarkers to appropriately choose those patients most likely to benefit from ICI are needed to improved efficacy and to avoid increased risk of toxicity for those who may not benefit (20). For example, eosinophils seem to be an interesting biomarker for the characteristics of severe checkpoint inhibitor pneumonitis (CPI). Indeed, Li et al. showed that the eosinophil percentage evolution in patients developing CPI under ICI could be associated with the diagnosis, prediction and prognosis of CIP and severe CIP. We have to help understand the underpinnings of molecular biology and interactions with the tumor immune environment to improve outcomes for NSCLC with genomic alterations (20). The host himself can interfere with ICI processing; indeed, numerous studies have evaluated the role of the microbiota in lung cancer and in the response to immunotherapy. Thus, a study from Ocáriz-Díez et al. presented the potential role of pulmonary or intestinal microbiota in improving ICI response with identification of microbial species associated with a favorable or unfavorable ICI response. Variability in response to immunotherapy may be partly explained by other patient characteristics. It was hypothesized that smoker patients would respond better to ICI than non-smokers. A meta-analysis of Zhao et al. brought together the results of 16 immunotherapy therapeutic trials allowing the analysis of smoking on survival. It seems to confirm the “benefit” of smoking history on the response to immunotherapy. Development of non-invasive biomarkers, such as circulating tumor DNA, can help guide the necessary duration of ICI treatment in metastatic patients and need for therapy in the curative setting (21). We cited PD-1, PD-L1, CTLA-4 immune checkpoints, but novel immune checkpoint targets are valued in the development of potentially more efficient ICI through combination therapy. We can consider combining ICI with each other and with different therapeutic modalities to improve the management of lung cancer.

The influence of immunotherapy in lung cancer has been very positive and the future impact is promising. ICI have become the standard of care treatment for most lung cancer patients, with undeniable improvement in survival and quality of life during treatment. Future immunotherapy treatments will further personalize the management of our lung cancer patients.

## Author contributions

KR, MF contributed to conception of the work. MF wrote the first draft of the editorial. KR wrote sections of the editorial. All authors contributed to editorial revision, read, and approved the submitted version.

## Funding

The project described was supported in part by Cedars-Sinai Cancer.

## Conflict of interest

The authors declare that the research was conducted in the absence of any commercial or financial relationships that could be construed as a potential conflict of interest.

## Publisher’s note

All claims expressed in this article are solely those of the authors and do not necessarily represent those of their affiliated organizations, or those of the publisher, the editors and the reviewers. Any product that may be evaluated in this article, or claim that may be made by its manufacturer, is not guaranteed or endorsed by the publisher.
